# Antiviral activity of dolutegravir in subjects with failure on an integrase inhibitor-based regimen: week 24 phase 3 results from VIKING-3

**DOI:** 10.7448/IAS.15.6.18112

**Published:** 2012-11-11

**Authors:** G Nichols, A Mills, R Grossberg, A Lazzarin, F Maggiolo, J Molina, G Pialoux, D Wright, M Ait-Khaled, J Huang, C Vavro, B Wynne, J Yeo

**Affiliations:** 1 GlaxoSmithKline, Research Triangle Park, USA; 2 Anthony Mills MD Inc, Los Angeles, USA; 3 Montefiore Medical Centre, New York, USA; 4 San Raffaele Scientific Institute, Milan, Italy; 5 Ospedale Riuniti, Bergamo, Italy; 6 Hospital Saint-Louis, Paris, France; 7 Hospital Tenon, Paris, France; 8 Central Texas Clinical Research, Austin, USA; 9 GlaxoSmithKline, London, UK; 10 GlaxoSmithKline, Mississauga, Canada; 11 GlaxoSmithKline, Philadelphia, USA

## Abstract

**Background:**

VIKING-3 aimed to examine efficacy and safety of dolutegravir (DTG) 50 mg twice daily in patients with resistance to multiple ARV classes, including integrase inhibitors (INI).

**Methods:**

RAL and/or EVG-resistant (current or historical) adult subjects with screening plasma HIV-1 RNA ≥500 c/mL and resistance to ≥2 other ART classes received open-label DTG 50 mg BID while continuing their failing regimen (without RAL/EVG). At Day 8 the background regimen was optimised and DTG continued. Activity of the optimized background regimen (OBR) was determined by Monogram Net Assessment. Primary endpoints were antiviral efficacy at Day 8 and Week 24.

**Results:**

183 subjects enrolled, 124 with INI-resistance at screening and 59 with historical (but no screening) resistance. Population was advanced: at BL, median CD4 140, prior ART 13 yrs, 56% CDC Class C; 79% had >2 NRTI, 75% >1 NNRTI, and 70% >2 PI resistance-associated mutations, and 61% had non-R5 HIV detected. Of the 114 subjects who had the opportunity to complete 24 weeks on study before data cutoff, 72 (63%) had <50 c/mL RNA at Week 24 (SNAPSHOT algorithm). Mean HIV RNA declined by 1.4 log_10_ c/mL (95% CI: 1.3, 1.5; p < 0.001) at Day 8; response differed by genotype pathway (Table).

In subjects with Q148 pathway mutations, virologic response decreased with increasing number of secondary mutations. Background overall susceptibility score (OSS) was not associated with Wk 24 response: % <50 c/mL were 83%, 63%, 59% and 69% for OSS 0, 1, 2 and >2, respectively. Discontinuations due to adverse events were uncommon (6/183, 3%); the most common drug-related AEs were diarrhoea, nausea and headache, each reported in only 5% of subjects.

**Conclusion:**

A majority of the highly treatment-experienced subjects in VIKING-3 achieved suppression with DTG-based therapy. Responses were associated with Baseline IN genotype but not OSS, highlighting the importance and independence of DTG antiviral activity. DTG had a low rate of discontinuation due to adverse events at 50 mg BID in this advanced patient population.

**Table:** 

Primary INI mutations at BL	N	Mean HIV RNA (log _10_) Change from BL (SD) at Day 8	%>1 log HIV RNA decline of <50 c/mL at Day 8
TOTAL	183	−1.4 (0.61)	82%
T66	1	−1.9	100%
Y143	28	−1.7 (0.42)	96%
N155	33	−1.4 (0.51)	82%
Q148 + ≤1 secondary mutation#	32	−1.1 (0.51)	69%
Q148 + ≥2 secondary mutations#	20	−1.0 (0.81)	48%
≥2 primary mutations	8	−1.4 (0.76)	75%
No primary mutations	60	−1.6 (0.55)	95%

# Key secondary mutations comprised G140_ACS, L741, E138_AKT.

